# Gut Microbiota and Brain Alterations after Refeeding in a Translational Anorexia Nervosa Rat Model

**DOI:** 10.3390/ijms24119496

**Published:** 2023-05-30

**Authors:** Stefanie Trinh, Vanessa Kogel, Lilly Kneisel, Elena Müller-Limberger, Beate Herpertz-Dahlmann, Cordian Beyer, Jochen Seitz

**Affiliations:** 1 Institute of Neuroanatomy, RWTH Aachen University, Wendlingweg 2, 52074 Aachen, Germany; ntrinh@ukaachen.de (S.T.); vanessa.kogel@rwth-aachen.de (V.K.); lilly.kneisel@rwth-aachen.de (L.K.); elena.mueller-limberger@uk-koeln.de (E.M.-L.); 2 Department of Child and Adolescent Psychiatry, Psychosomatics and Psychotherapy, RWTH Aachen University, Neuenhofer Weg 21, 52074 Aachen, Germany; bherpertz@ukaachen.de (B.H.-D.); jseitz@ukaachen.de (J.S.)

**Keywords:** activity-based anorexia, anorexia nervosa, astrocyte, microbiome, microbiota–gut–brain axis

## Abstract

The gut microbiota composition is causally involved in the regulation of body weight. Through the gut–brain axis, microbiota play a role in psychiatric disorders including anorexia nervosa (AN). Previously, we showed microbiome changes to be associated with brain volume and astrocyte reductions after chronic starvation in an AN animal model. Here, we analyzed whether these alterations are reversible after refeeding. The activity-based anorexia (ABA) model is a well-established animal model that mimics several symptoms of AN. Fecal samples and the brain were analyzed. Like previous results, significant alterations in the microbiome were observed after starvation. After refeeding, including the normalization of food intake and body weight, α- and β-diversity, as well as the relative abundance of specific genera, were largely normalized in starved rats. Brain parameters appeared to normalize alongside microbial restitution with some aberrations in the white matter. We confirmed our previous findings of microbial dysbiosis during starvation and showed a high degree of reversibility. Thus, microbiome alterations in the ABA model appear to be mostly starvation-related. These findings support the usefulness of the ABA model in investigating starvation-induced effects on the microbiota–gut–brain axis to help comprehend the pathomechanisms of AN and potentially develop microbiome-targeted treatments for patients.

## 1. Introduction

Anorexia nervosa (AN) is one of the most severe eating disorders. It is characterized by a disturbed body image and an intense fear of gaining weight, which motivates food restriction and occasionally other weight loss behaviors such as excessive physical activity [1]. AN is significantly more prevalent in girls and young women and has the highest mortality rate of all mental disorders and an exceptionally high relapse rate [2,3]. Lately, associations between gut microbiota and psychiatric illnesses have been intensively studied and provided evidence for major perturbations in the microbial composition in, for example, patients with autism spectrum disorders [4] or major depression [5,6]. Furthermore, several studies have proposed a considerable intestinal dysbiosis in AN, which was only partly reversible with body weight gain [7,8,9,10]. In addition, processes that might be at play in the pathophysiology of AN (e.g., appetite and body weight regulation, metabolic and immunological processes) were shown to be affected by the gut microbiome [11,12,13,14]. A small number of studies have also shown clear alterations in the gut microbiota composition in rodents because of food restriction [15,16,17]. An initial preclinical study transferred fecal samples from patients with AN into germ-free (gf-) mice, so-called fecal microbiota transplantation, and observed reduced body weight gain in the offspring after six weeks because of reduced food intake compared with mice that received fecal samples from healthy controls [18]. Similarly, stool transplantation from malnourished, underweight children in Malawi and Bangladesh into gf-mice lead to reduced growth in the animals [19]. The AN-transplanted animals also showed increased obsessive- and anxiety-like behavior after the transplantation, examples of microbiota–gut–brain interactions. Supplementation with a probiotic bacterium, *Bacteroides vulgatus*, ameliorated these behaviors [18]. In contrast, a study by Glenny et al. (2021) was not able to observe any changes after four weeks in body weight or feeding behavior after fecal microbiota transplantation from patients with AN [20].

In our own study, we previously showed associations between gut microbiota changes and brain parameters such as brain volume loss and reduced astrocyte cell numbers; for example, lower brain volumes were associated with higher microbiota diversity found in food-restricted rats [17]. A substantial decline of gray brain matter is associated with starvation and a poor prognosis in patients with AN [21,22], a process that might involve the loss of astrocytes, as proposed by translational animal studies [17,23,24]. 

The purpose of the present study was to follow up on previously observed increases in bacterial richness (α-diversity) and differences in bacterial composition (β-diversity) during chronic food restriction in the activity-based anorexia (ABA) model. We aimed to precisely analyze whether acute starvation leads to such alterations and whether refeeding and body weight restoration reverse these effects on the gut microbiome and brain parameters. 

## 2. Results

### 2.1. Changes in Body Weight, Food Intake, and Running-Wheel Activity

**Figure 1:**
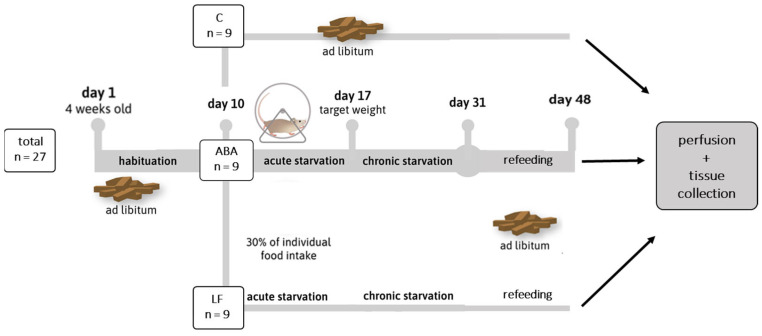
Study design. C = control animals with ad libitum food access but no running-wheel access; ABA = animals with limited food and running-wheel access; LF = animals with limited food but no running-wheel access.

**Figure 2:**
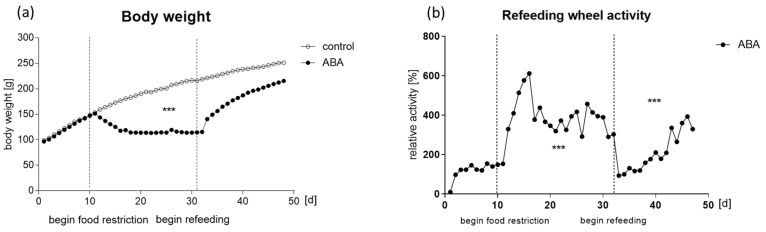
Body weight and activity profile. (**a**) Mean body weight in grams per day; (**b**) mean running-wheel activity in % normalized to the average running-wheel activity of the habituation period (days 1–10). ABA = activity-based anorexia. *** *p* ≤ 0.001.

Figure 1 displays the study protocol. As expected, the body weight of all animals increased during the habituation period as the young animals were still growing. From day 10 onward, when food restriction was applied to the ABA group, the average body weight of these animals decreased continuously until they lost 25% of their initial body weight after, on average, 5.89 days (*p* = 0.0001; Figure 2a). After approximately two weeks of additional chronic starvation, ABA and LF rats again had free access to food during the refeeding period (Supplementary Figure S1a,b), leading to an immediate increase in body weight (Figure 2a). Even though the body weight of the ABA animals did not reach the exact same level as that of the control group at the end of the experiment, there was no statistically significant difference anymore. Similar effects could be observed for LF animals (Supplementary Figure S1c). Even though no control group with a running-wheel but no food restriction was available, the activity levels of the ABA animals could be observed over time. Running-wheel activity was constantly low during the habituation period in the ABA animals (Figure 2b). During the food starvation period, a considerable increase in physical activity was obvious, which is often considered food-anticipatory activity or food-seeking behavior [25,26]. At the end of the acute starvation period, the activity levels of the rats lowered again, probably to prevent exhaustion. Refeeding caused an initial lapse in running activity, as the animals dramatically increased feeding only to slowly normalize again during the rest of the refeeding period (Figure 2b). 

### 2.2. Changes in Microbial Richness and Composition

**Figure 3:**
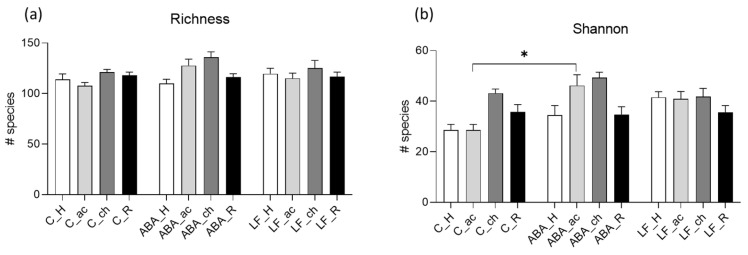

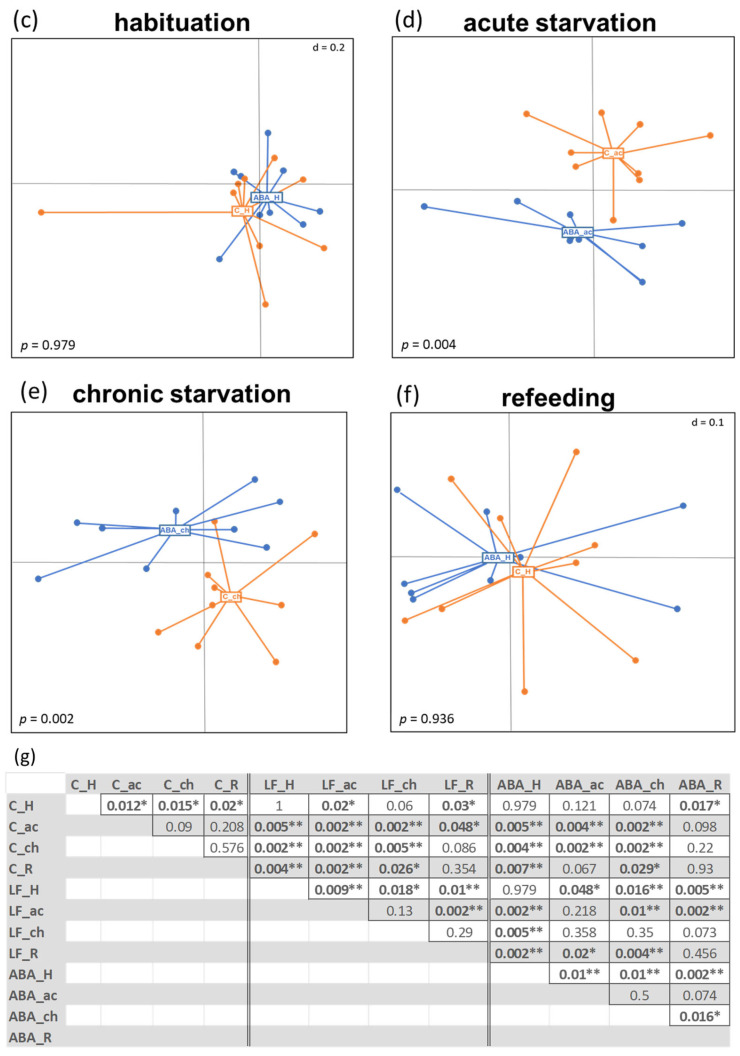
α-diversity and β-diversity metrics. (**a**) Species richness and (**b**) Shannon effective count as α-diversity measurements. (**c**–**f**) Generalized UniFrac distances as β-diversity measurement in a multidimensional, non-scaling plot at different time points (habituation, acute starvation, chronic starvation, refeeding). (**g**) *p*-values of generalized UniFrac distances comparing C, ABA, and LF groups at different time points. Statistically significant *p*-values are marked with and asterix and written in bold. C = control, ABA = activity-based anorexia, LF = limited food, H = habituation, ac = acute starvation, ch = chronic starvation, R = refeeding. * *p* ≤ 0.05, ** *p* ≤ 0.01.

In accordance with our previous study, the species richness and Shannon index were nominally increased during acute and chronic starvation in food-restricted rats compared with normally fed controls at the same time point, reaching significance for the Shannon index in acutely starved animals (Figure 3a,b). After refeeding, the α-diversity parameters of the LF/ABA rats showed similar levels to the control rats (Figure 3a,b). 

Comparing the microbial composition (β-diversity) during the acute and chronic starvation phases, a significant difference between the ABA and control group could be observed (acute starvation: *p* = 0.004, chronic starvation: *p* = 0.002; Figure 3d,e). No significant differences were detected during the habituation and refeeding phase, thus pointing to normalization after weight recovery (Figure 3c,f). Similar results were observed when comparing the limited food and control groups (Supplementary Figure S3). Interestingly, on a longitudinal scale, the microbiota composition in the ABA animals (and the LF animals) at habituation differed significantly from the microbiota at refeeding (Figure 3g). However, since this effect was also present in the control animals, it is probably a maturational effect. All *p*-values for the β-diversity measurement comparing controls with the LF and ABA groups during the different measurement time points are provided in Figure 3g.

**Figure 4:**
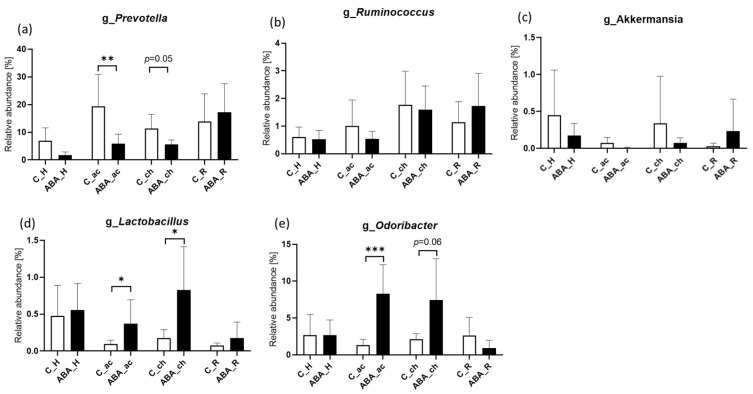
Relative abundance of specific genera. Relative abundance in % of the genus: (**a**) *Prevotella*, (**b**) *Ruminococcus*, (**c**) *Akkermansia*, (**d**) *Lactobacillus*, and (**e**) *Odoribacter* at different measure time points (habituation, acute starvation, chronic starvation, refeeding). C = control, ABA = activity-based anorexia, H = habituation, ac = acute starvation, ch = chronic starvation, R = refeeding. * *p* ≤ 0.05, ** *p* ≤ 0.01, *** *p* ≤ 0.001.

Most of the 21 genera meeting the inclusion criteria for the analysis described in the method section (Supplementary Table S1) did not show any clear changes during acute and chronic starvation, nor differences after refeeding. Focusing on five genera that showed alterations during chronic starvation in our previous study [17], we found a similar picture in the current study for both acute and chronic starvation. The relative abundance of *Prevotella* and *Ruminococcus* decreased during starvation (Figure 4a,b), while the relative abundance of *Lactobacillus* and *Odoribacter* significantly increased during starvation, comparing the ABA animals with the controls (Figure 4d,e). Similar effects were observed comparing the LF animals and controls (Supplementary Figure S4). The previously found increase in the relative abundance of *Akkermansia* could not be confirmed in ABA rats (Figure 4c) but showed a trend toward an increase in LF rats compared with the controls (Supplementary Figure S3c). After refeeding, no more differences could be found between the control group and the food-restricted rats (LF and ABA).

### 2.3. Changes in Brain Volume, GFAP+ Cell Numbers, and mRNA Expression 

**Figure 5:**
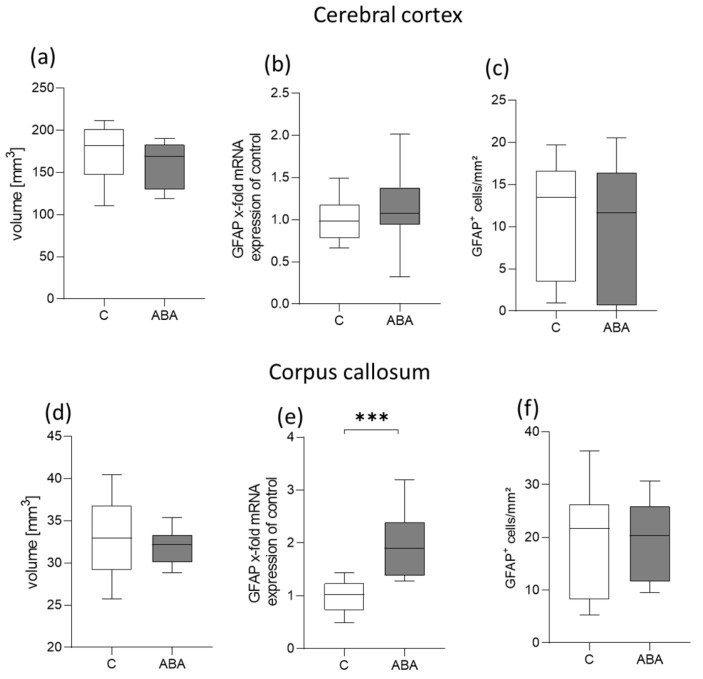
Brain alterations after refeeding. (**a**) Mean brain volume in mm^3^ in the cerebral cortex, (**b**) mRNA expression of *GFAP* in the cerebral cortex, (**c**) mean number of GFAP+ cells per mm^2^ in the cerebral cortex, (**d**) mean brain volume in mm^3^ in the corpus callosum, (**e**) mRNA expression of *GFAP* in the corpus callosum, (**f**) mean number of GFAP+ cells per mm^2^ in the corpus callosum. GFAP = glial fibrillary acidic protein, C = control, ABA = activity-based anorexia. *** *p* ≤ 0.001.

Brain volume, the average number of GFAP+ astrocytes, and the quantification of *GFAP* transcripts were measured in the cerebral cortex and the corpus callosum. After body weight recovery, only a few differences were apparent between the ABA and control rats or LF and control rats (Figure 5 and Supplementary Figure S5). In the ABA rats, a significant increase in the mRNA expression of *GFAP* in the corpus callosum was examined after refeeding (*p* = 0.0008; Figure 5e). In the LF rats, a significant increase in the mRNA expression of *GFAP* in the corpus callosum (*p* = 0.04) and cerebral cortex (*p* = 0.02) was also observed (Supplementary Figure S5b,e). In addition, a significant increase in GFAP+ astrocytes in the corpus callosum of the LF rats was counted (*p* = 0.04; Supplementary Figure S5f). 

After body weight restoration, no more relevant correlations between alterations in the gut microbiome (α- and β-diversity and relative abundances of taxa) and the brain were evident.

## 3. Discussion

To the best of our knowledge, this study is the first to explore the gut microbiome in a preclinical animal model after body weight restoration. In the present study, we confirmed that food restriction significantly and reversibly affects gut microbial richness and composition. As most outcome parameters were altered in a similar pattern in both food-restricted groups (LF and ABA), it could be assumed that food restriction is the main driving factor for these microbiome alterations rather than running-wheel activity. Bacterial richness tended to increase during acute and chronic food reduction and normalized to initial levels after refeeding compared with the controls at the respective time points, especially in the ABA group (see Figure 3b). This stands in contrast with studies on patients with AN, where reduced or non-altered α-diversity was found in acutely ill patients with an increase after weight gain [8,9,10,26]. This contradictory finding in our animal model could be explained by niche-inhabiting bacterial species that are only present in a small number of control animals. These specific bacteria could have a selection advantage in the case of food starvation and thus become apparent in α-diversity measures. Mucin-degrading bacteria such as *Akkermansia* might be an example, and they were found to accumulate in our limited-food animals (see Supplementary Figure S3c) and have been identified in acutely starved patients with AN [10,17]. Likewise, β-diversity was significantly different between the food-restricted groups compared with the controls during acute and chronic starvation and normalized after refeeding. This fits the picture of short-term refeeding leading to a partial rehabilitation of gut microbiota alterations in patients with AN [7,9,26]. However, contrary to our experiment, complete normalization after short-term weight recovery was not evident in adults and adolescent patients with AN [7,9,26]. As most patients still have a lower body mass index and different dietary habits at discharge in comparison with healthy controls, this could explain the remaining differences. Further, we assume that the timeframe used in these clinical studies was too short to reach complete recovery. On the other hand, these findings from patient studies might be associated with the ongoing causal effects of gut microbiota favoring illness maintenance, as suggested by fecal microbiota transplantation studies, where transplanting feces from patients with AN (or malnourished children) leads to reduced weight gain in germ-free mice [18,19]. 

Only a few genera showed significant alterations in their relative abundance during starvation. Similar to our previous study and the study by Hata and colleagues (2019), we detected increased levels of *Odoribacter* in food-restricted animals [17,18]. Hata et al. found an association between the relative abundance of *Odoribacter* and food efficiency in starved mice [18]. Furthermore, *Odoribacter* produces short-chain fatty acids such as butyrate, which is known to stimulate the production of mucus, provide nutrients for gut enterocytes, and decrease gut permeability. Thus, the detected increase in *Odoribacter* in all animals with lowered body weight might be an adaptation mechanism to protect the gut wall integrity, which is assumed to be impaired because of starvation; see Chen et al., 2017 and Yan et al., 2017. Comparable to a study in female patients with AN, the relative abundance of *Prevotella* was significantly decreased in food-restricted animals in the present study [27]. In contrast, increased levels of *Prevotella* were measured in an ABA model using male rats [16]. A variety of subtypes of *Lactobacillus* are commonly used for probiotic supplementation, which is known to exert positive effects on general health status and mood during application [28]. As we detected a significant rise in *Lactobacillus* in ABA rats, we propose a protection mechanism induced by continuous food starvation. 

As seen before, reduced brain volumes and GFAP+ astrocyte levels seem to be mostly reversible because of increased food intake after a starvation period [23], fitting well with findings of mostly reversible brain volume reduction in patients with AN [22]. The remaining differences, especially in astrocytes located in the white brain matter (corpus callosum) might be because of ongoing regeneration effects in this tissue in response to astrocyte reductions in the ABA animal model. Furthermore, previous animal and human studies have shown lasting differences especially in white brain matter after refeeding, pointing to differing mechanisms underlying gray and white matter brain volume reduction and rehabilitation [22,23]. A longer refeeding time might be necessary to show complete normalization. No germane relationships between brain and gut microbiota alterations were obvious after refeeding, which might correspond to high variability in brain tissue and gut microbiota in a healthy state. However, because of the low number of samples, nine per group, small effects might remain undetected. 

In summary, the effects of starvation on the gut microbiota diversity and composition seem largely reversible in the animal model when allowing ad libitum feeding after a one-time ABA exposure. 

The ABA model is a well-established translational model for AN that has been proven to function as a useful tool mimicking several aspects of human AN in rodents [29]. Besides somatic symptoms such as body weight loss, hyperactivity, amenorrhea, and brain volume loss, behavioral aspects, for example, increased anxiety-like behavior and memory/visual–spatial cognition impairments and, recently, gut microbiome dysbiosis, showed the value of using the ABA model [29,30,31]. Moreover, this allows us to monitor the same animals at different time points (before starvation vs. during acute and chronic starvation vs. after refeeding) under extreme standardized conditions (food, environmental factors, etc.), which increases the internal validity of the model. Still, observed discrepancies between study results from rodents versus study results from humans should be noted; for example, the ease with which ABA animals restore their body weight may reflect rodent versus human-specific variations and the fact that AN is a multifactorial disease. Fecal microbiota transplantations from patients with AN into gf-rodents to establish a “humanized” rodent model represent possible next steps in understanding the causal role of the microbial ecosystem in eating disorders such as AN. Studies using nutritional supplements such as omega-3 fatty acids or probiotics may contribute to developing new intervention strategies that target the microbiome and complement current treatment options.

## 4. Materials and Methods

### 4.1. Animals

Twenty-seven female Wistar rats (3 weeks old) were commercially purchased (RjHan:WI; Janvier, Hannover, Germany). All animals were housed at controlled room temperature (23 °C) and humidity (55%) and on a 12 h light/dark cycle (lights on at 7 am) in type IV cages (Polysulfone, Tecniplast GmbH, Hohenpeißenberg, Germany). The animal facility was specifically pathogen-free in accordance with DIN ISO 9001:2008. The animal procedure was permitted by the Governmental Animal Care and Use Committee, LANUV North Rhine Westphalia (Landesamt für Umwelt, Natur und Verbraucherschutz, Recklinghausen, Germany) with approval number 81-02.04.2021.A183. All experiments were performed in accordance with German legislation governing animal studies following the Guide for the Care and Use of Laboratory Animals (NIH publication, 8th edition, 2011) and the 2010/63/EU Directive on the protection of animals used for scientific purposes (*Official Journal of the European Union*, 2010).

### 4.2. Study Design

A modified version of the ABA model was established in our laboratory, described in detail previously [32]. In accordance with our previous study [17], all animals had a 10-day acclimatization period to habituate to the individual housing conditions and the running-wheel, if applicable. During the habituation period, all animals had ad libitum access to food and water. Subsequently, the animals were randomly assigned to three groups (n = 9 each): control animals with ad libitum food access but no running-wheel access (C), animals with limited food but no running-wheel access (LF), and animals with limited food and running-wheel access (activity-based anorexia, ABA) (Figure 1). On day 10, daily food access of the LF and ABA rats was reduced to 30% of the average food intake during habituation (defined as the acute starvation period) until the rats lost 25% of their body weight (defined as the target weight). When the animals reached the target weight, the amount of food was adjusted daily for each individual animal to ensure a stable but low body weight until day 30 (defined as the chronic starvation period). Following day 31, a refeeding phase with ad libitum access to food for 14 days was added to the protocol. The control group continuously received ad libitum food until the end of the protocol. The running-wheel of the control and the limited food (LF) group was blocked in the cage in such a way that it did not turn. The body weight; the food intake; and, if applicable, the running-wheel activity were recorded every day at 12 a.m. A graphical representation of the study design is shown in Figure 1. 

### 4.3. Microbiome Analysis

Per-animal fecal samples were collected at the following time points: (1) after habituation (H, day 9), (2) after acute starvation (ac, day 17), (3) after chronic starvation (ch, day 31), and (4) after refeeding (R, day 48) or at corresponding time points for the control rats. All samples were freshly collected in sterile plastic microtubes (1.5 mL, Sarstedt, Nümbrecht, Germany) and immediately stored at −80 °C. High-throughput 16S rRNA gene amplicon sequencing of the V3/V4 region was performed, as previously described [17]. The obtained sequencing data were processed based on the UPARSE approach using the IMNGS platform [33]. In brief, the inclusion criteria for analysis were as follows: after de-multiplexing, all sequences were trimmed to the first base with a quality score lower than 3. Only sequences between 300 and 600 nucleotides were included in the analysis. Operational taxonomic units (OTUs) were clustered in a sequence similarity of 97%, and only those with a relative abundance higher than 0.5% in at least one sample were included. All genera meeting these inclusion criteria are represented in Supplementary Table S1. Taxonomies were assigned using the RDP classifier. Downstream analysis was performed using Rhea in the R programming environment [34]. For α-diversity, the species richness, Shannon and Simpson index, and Shannon and Simpson effective count were calculated. Because of the strong weight toward high relative abundances for the Simpson index (Supplementary Figure S2), the species richness and Shannon effective counts are preferred. For β-diversity measurements, generalized UniFrac distances as a balanced version between weighted and unweighted UniFrac distances, which are sensitive to dominant and rare species, respectively, were computed. α-diversity assesses the number of different species within a sample, while β-diversity assesses microbiome similarities between individual groups. The following sequencing data are presented at the genus level to avoid taxonomic misclassification because of unknown or incompletely classified species.

### 4.4. Brain Volume Measurements

After transcardial perfusion with an artificial cerebrospinal fluid solution, the brains were immediately extracted on ice and divided at the interhemispheric cleft. The right hemisphere of each rat was post-fixated with paraformaldehyde (3.7%) and cryo-protected via dehydration with 10 and 30% sucrose solutions. Afterward, the hemispheres were embedded in an optimal cutting temperature medium (Sakura Finetek, Alphen aan den Rijn, the Netherlands) and cut frontally into a series of 100 µm sections on a cryostat at −20 °C (Leica CM 3050S, Nussloch, Germany). Every second slice was stained with hematoxylin–eosin. Slices were digitalized, and regions of interest (cerebral cortex: Bregma 5.2–9.8; corpus callosum: Bregma 3.7–8.0) were individually marked on each image with the polygon selection tool in the ImageJ software (1.48 v, Wayne Rasband, National Institutes of Health, Bethesda, MD, USA). The Cavalieri method (multiplying the individual areas by the slice thickness and adding up these results per hemisphere) was used to calculate the volumes of the regions of interest. The investigator was blinded to the group affiliations of the samples. 

### 4.5. Immunohistochemistry 

In addition, a series of 20 µm sections at Bregma −2.30 were cut. Two slices per animal were immunohistochemically stained with a goat anti-GFAP antibody (glial fibrillary acidic protein, sc-6170, Santa Cruz, CA, USA). The antibody was used in a concentration of 1:750 using standard procedures, described in detail elsewhere [35]. All slices were digitalized and analyzed with the ImageJ software (1.48 v, Wayne Rasband, National Institutes of Health, Bethesda, MD, USA). Two blinded and independent observers counted all GFAP+ cells containing a visible nucleus in the regions of interest (cerebral cortex and corpus callosum). The results are expressed as the mean number of cells/mm^2^. 

### 4.6. Reverse Transcription and Real-Time Polymerase Chain Reaction (rtPCR)

From the left hemisphere, RNA was isolated using PeqGold RNA Trifast (Peqlab, Erlangen, Germany), as previously described [36]. Afterward, complementary DNA were reversely transcribed from the isolated RNA. The relative expression of the mRNA was analyzed using rtPCR. The ratio between the reference gene (Cyclophilin A, sense: 5′-GGCAAATGCTGGACCAAACAC, antisense: 5′-TTAGAGTTGTCCACAGTCGGG AGATG) and the gene of interest (GFAP, sense: 5′-AGAAAACCGCATCACCATT, antisense: 5′-GCACACCTCACATCACATCC) was calculated and described as the fold change relative to the control group. 

### 4.7. Statistical Analysis

Body weight, food intake, and running-wheel activity were averaged per group and separated into the different phases of the experimental protocol (habituation: days 1–10, starvation: days 11–31, refeeding: days 32–48). All parameters were normalized to the baseline measured during the habituation period, and differences between the control and ABA group were evaluated with Student’s *t*-test. Differences over time in the running-wheel activity of the ABA group were evaluated in a repeated-measure analysis of variances (ANOVA) with time as the within-subject factor. Differences in the α-diversity (richness and Shannon Index) were evaluated in an ANOVA per time point with Bonferroni correction for multiple comparisons. Differences in the β-diversity measurement (generalized UniFrac distance) were calculated using ANOVA on ranks, corrected for multiple comparisons according to the Benjamini–Hochberg method, and graphically depicted in a multi-dimensional non-scaling plot. Correlation analyses between gut microbiota and brain parameters were calculated via Spearman correlation and corrected for multiple testing using the false discovery rate. For the sake of simplicity, most results of the LF group are provided in the Supplementary Materials. Statistical analyses were performed with SPSS Statistics version 25 for Windows (IBM, Chicago, IL, USA), and the significance level for all comparisons was set to 5%.

## 5. Conclusions

In conclusion, we confirmed and more deeply characterized our previous findings on microbial dysbiosis during chronic starvation, expanded them to acute starvation, and demonstrated their reversibility upon refeeding. We believe that these study results contribute to further validating the ABA model and will enable future studies to comprehend the exact and causal involvement of the gut microbiome in AN and how to manipulate it to potentially develop new treatment possibilities for patients with AN.

## Data Availability

The data presented in this study are available upon request from the corresponding author.

## Supplementary Materials

The following supporting information can be downloaded at https://www.mdpi.com/article/10.3390/ijms24119496/s1.
