# Supplementary material for: Gut Microbiota and Brain Alterations after Refeeding in a Translational Anorexia Nervosa Rat Model
Source: Int J Mol Sci. 2023 May 30;24(11):9496. doi: 10.3390/ijms24119496 (PMC10253567; doi:10.3390/ijms24119496)
Supplement: Supplementary file 1 [file ijms-24-09496-s001.zip › ijms-2399563-supplementary.pdf]

International Journal of *Molecular Sciences*

MDPI

Article

Gut Microbiota and Brain Alterations after Refeeding in a Translational Anorexia Nervosa Rat Model

Stefanie Trinh [1], Vanessa Kogel [1], Lilly Kneisel [1], Elena Müller-Limberger [1], Beate Herpertz-Dahlmann [2], Cordian Beyer [1,*] and Jochen Seitz [2]

[1] Institute of Neuroanatomy, RWTH Aachen University, Wendlingweg 2, 52074 Aachen, Germany; ntrinh@ukaachen.de (S.T.); vanessa.kogel@rwth-aachen.de (V.K.); lilly.kneisel@rwth-aachen.de (L.K.); elena.mueller-limberger@uk-koeln.de (E.M.-L.)

[2] Department of Child and Adolescent Psychiatry, Psychosomatics and Psychotherapy, RWTH Aachen University, Neuenhofer Weg 21, 52074 Aachen, Germany; bherpertz@ukaachen.de (B.H.-D.); jseitz@ukaachen.de (J.S.)

* Correspondence: cbeyer@ukaachen.de

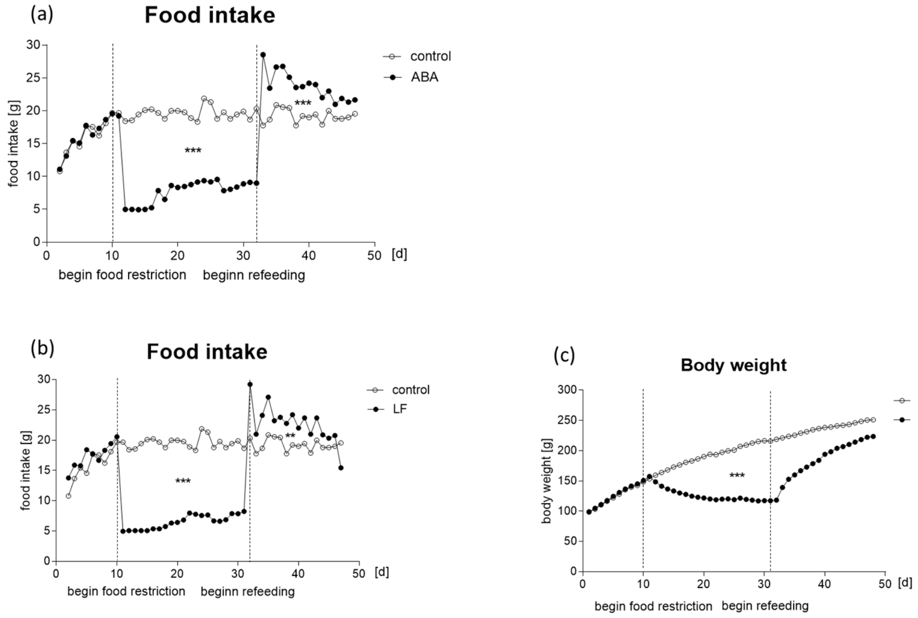

Supplementary Figure S1: Food intake and body weight profile. (**a**) mean food intake in grams per day for ABA group, (**b**) mean food intake in grams per day for LF group, (**c**) Mean body weight in grams per day for LF group. C = control, ABA = activity-based anorexia, LF = limited food ** $p \leq 0.01$, *** $p \leq 0.001$.

Citation: Trinh, S.; Kogel, V.; Kneisel, L.; Müller-Limberger, E.; Herpertz-Dahlmann, B.; Beyer, C.; Seitz, J. Gut Microbiota and Brain Alterations after Refeeding in a Translational Anorexia Nervosa Rat Model. *Int. J. Mol. Sci.* **2023**, *24*, 9496. https://doi.org/10.3390/ijms24119496

Academic Editors: Dan Cristian Vodnar and Ashu Johri

Received: 29 April 2023
Revised: 23 May 2023
Accepted: 27 May 2023
Published: 30 May 2023

Copyright: © 2023 by the authors. Submitted for possible open access publication under the terms and conditions of the Creative Commons Attribution (CC BY) license (https://creativecommons.org/licenses/by/4.0/).

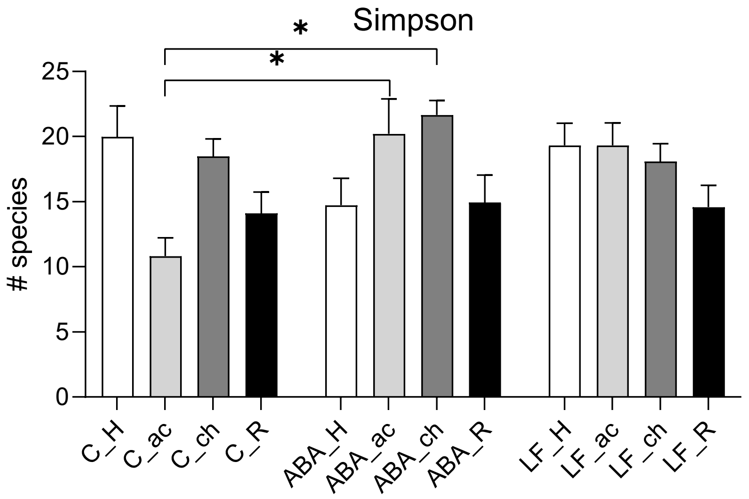

Supplementary Figure S2: Simpson effective count as α-diversity measurement. C = control, LF = limited food, H = habituation, ac = acute starvation, ch = chronic starvation, R = refeeding. * $p \leq 0.05$.

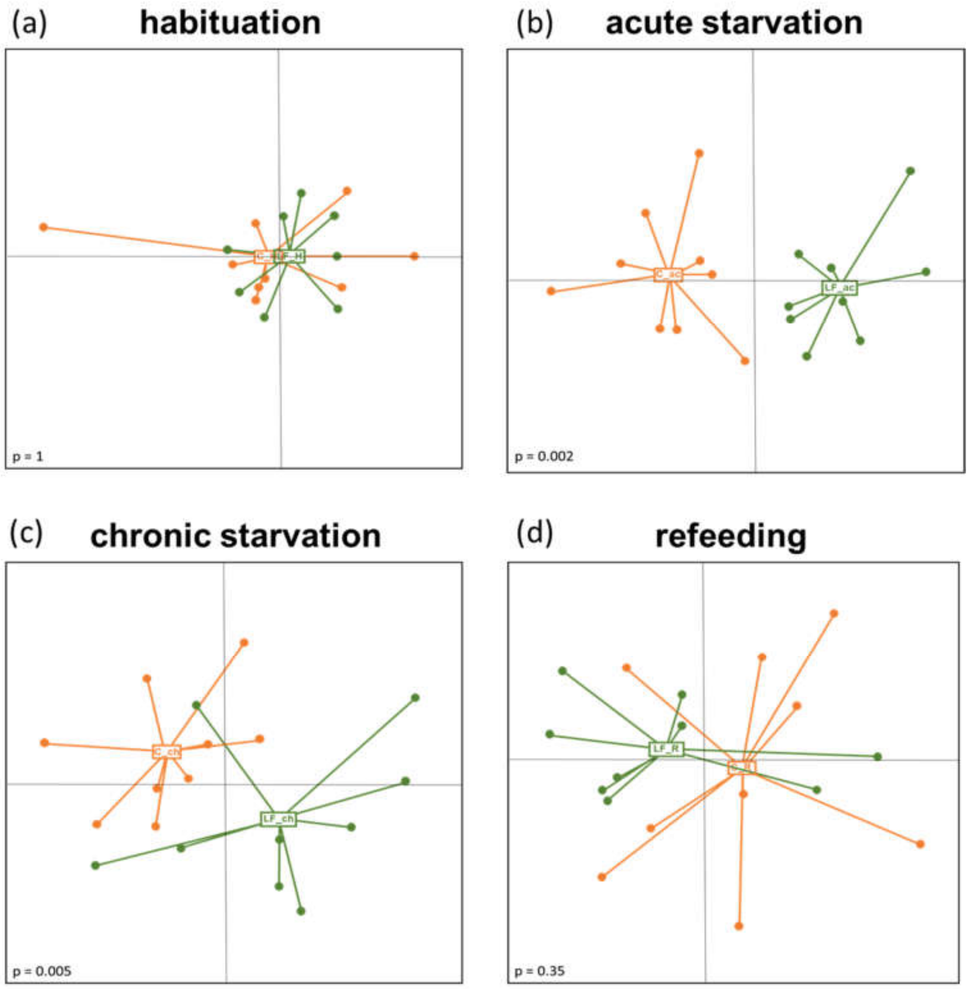

Supplementary Figure S3: β-diversity metrics in limited food vs. control group.

(**a**), (**b**), (**c**), and (**d**) generalized UniFrac distances as β-diversity measurement in a multidimensional non-scaling plot at different time points (habituation, acute starvation, chronic starvation, refeed-ing). C = control, LF = limited food, H = habituation, ac = acute starvation, ch = chronic starvation, R = refeeding.

Supplementary Table S1: All genera meeting inclusion criteria

21 genera that meet inclusion criteria for analysis described in the microbiome analysis section.

**Akkermansia**
**Alistipes**
**Alloprevotella**
**Bacteroides**
**Blautia**
**Butyricicoccus**
**Clostridium_IV**
**Clostridium_XI**
**Clostridium_XlVa**
**Clostridium_XlVb**
**Clostridium_XVIII**
**Flavonifractor**
**Hydrogenoanaerobacterium**
**Lactobacillus**
**Odoribacter**
**Oscillibacter**
**Prevotella**
**Pseudoflavonifractor**
**Ruminococcus**
**Sporobacter**
**Turicibacter**

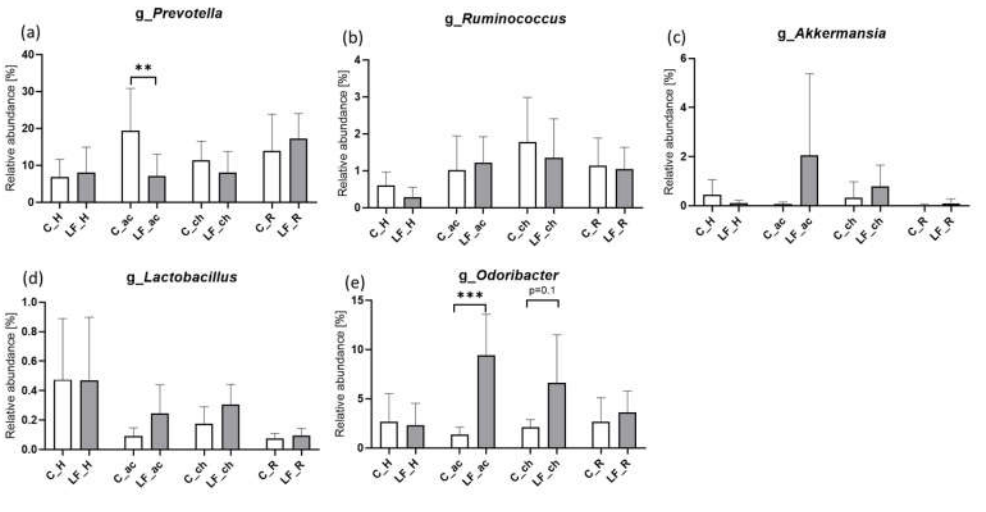

Supplementary Figure S4: Relative abundance of specific genera in limited food group

Relative abundance in % of the genus (**a**) *Prevotella*, (**b**) *Ruminococcus*, (**c**) *Akkermansia*, (**d**) *Lactobacil-lus*, and (e) *Odoribacter* at different measure time points (habituation, acute starvation, chronic star-vation, refeeding). C = control, LF = limited food, H = habituation, ac = acute starvation, ch = chronic starvation, R = refeeding. ** $p \leq 0.01$, *** $p \leq 0.001$.

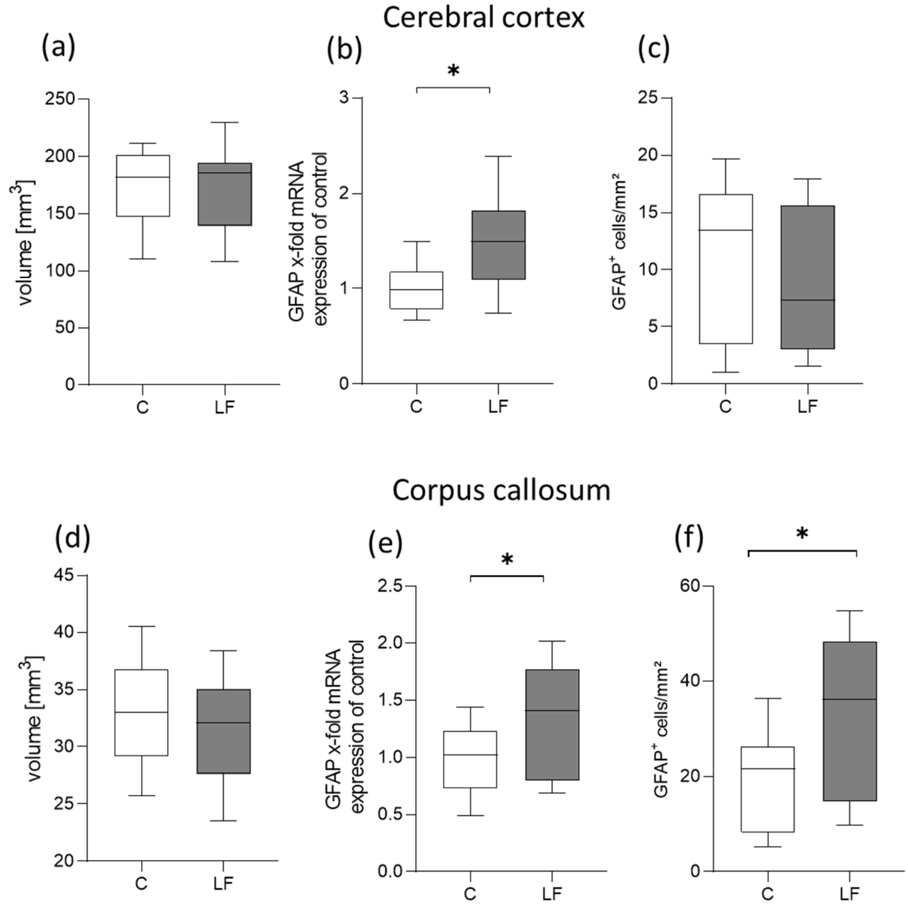

Supplementary Figure S5: Brain alterations after refeeding in limited food group

(**a**) Mean brain volume in mm³ in the cerebral cortex, (**b**) mRNA expression of GFAP in the cerebral cortex, (**c**) mean number of GFAP+ cells per mm² in the cerebral cortex, (**d**) mean brain volume in mm³ in the corpus callosum, (**e**) mRNA expression of GFAP in the corpus callosum, (**f**) mean number of GFAP+ cells per mm² in the corpus callosum. GFAP = glial fibrillary acidic protein, C = control, LF = limited food. * $p \leq 0.05$.
